# Evaluation of the effect of *Lactobacillus reuteri* V3401 on biomarkers of inflammation, cardiovascular risk and liver steatosis in obese adults with metabolic syndrome: a randomized clinical trial (PROSIR)

**DOI:** 10.1186/s12906-018-2371-x

**Published:** 2018-11-20

**Authors:** Carmen Tenorio-Jiménez, María José Martínez-Ramírez, Mercedes Tercero-Lozano, Carmen Arraiza-Irigoyen, Isabel Del Castillo-Codes, Josune Olza, Julio Plaza-Díaz, Luis Fontana, Jairo H. Migueles, Mónica Olivares, Ángel Gil, Carolina Gomez-Llorente

**Affiliations:** 1 Unidad de Gestión Clínica de Endocrinología y Nutrición. Complejo Hospitalario de Jaén, Jaén, Spain; 2 Departamento de Ciencias de la Salud. Facultad de Ciencias de la Salud, Universidad de Jaén, Jaén, Spain; 3 Unidad de Gestión Clínica de Aparato Digestivo. Complejo Hospitalario de Jaén, Jaén, Spain; 4 Department of Biochemistry and Molecular Biology II, University of Granada, Granada, Spain; 5 Institute of Nutrition and Food Technology “José Mataix”, Centre of Biomedical Research, University of Granada, Campus de la Salud, 18100 Armilla, Granada, Spain; 6 Instituto de Investigación Biosanitaria, ibs.GRANADA, Granada, Spain; 7 CIBER Physiopathology of Obesity and Nutrition CB12/03/30028 (CIBEROBN), Instituto de Salud Carlos III, Madrid, Spain; 8 PROFITH “PROmoting FITness and Health through physical activity” Research Group, Department of Physical Education and Sports, Faculty of Sport Sciences, University of Granada, Ctra. Alfacar s/n, 18011 Granada, Spain; 9 Biosearch Life, Granada, Spain

**Keywords:** Human adults, Insulin resistance syndrome, *Lactobacillus reuteri* V3401, Non-alcoholic fatty liver disease, Obesity, Probiotics

## Abstract

**Background:**

Obesity is characterized by increased fat mass and is associated with the development of insulin resistance syndrome (IRS), usually known as metabolic syndrome. The alteration of the intestinal microbiota composition has a role in the development of IRS associated with obesity, and probiotics, which are live microorganisms that confer a health benefit to the host, contribute to restore intestinal microbiota homeostasis and lower peripheral tissue insulin resistance. We aim to evaluate the effects of the probiotic strain *Lactobacillus reuteri* (*L. reuteri)* V3401 on the composition of intestinal microbiota, markers of insulin resistance and biomarkers of inflammation, cardiovascular risk, and hepatic steatosis in patients with overweight and obesity exhibiting IRS.

**Methods/design:**

We describe a randomized, double-blind, crossover, placebo-controlled, and single-centre trial. Sixty participants (aged 18 to 65 years) diagnosed with IRS will be randomized in a 1:1 ratio to receive either a daily dose of placebo or 5 × 10^9^ colony-forming units of *L. reuteri* V3401. The study will consist of two intervention periods of 12 weeks separated by a washout period of 6 weeks and preceded by another washout period of 2 weeks. The primary outcome will be the change in plasma lipopolysaccharide (LPS) levels at 12 weeks. Secondary outcomes will include anthropometric parameters, lipid profile, glucose metabolism, microbiota composition, hepatic steatosis, and inflammatory and cardiovascular biomarkers. Blood and stool samples will be collected at baseline, at the midpoint (only stool samples) and immediately after each intervention period. Luminex technology will be used to measure interleukins. For statistical analysis, a mixed ANOVA model will be employed to calculate changes in the outcome variables.

**Discussion:**

This is the first time that *L. reuteri* V3401 will be evaluated in patients with IRS. Therefore, this study will provide valuable scientific information about the effects of this strain in metabolic syndrome patients.

**Trial registration:**

The trial has been retrospectively registered in ClinicalTrials.gov on the 23rd November 2016 (ID: NCT02972567), during the recruitment phase.

**Electronic supplementary material:**

The online version of this article (10.1186/s12906-018-2371-x) contains supplementary material, which is available to authorized users.

## Trial status

Currently, the trial is ongoing and the analyses of the data and biomarkers continuing.

## Background

Obesity is a chronic, low-grade systemic inflammatory disease that is complex and multifactorial, with both genetic and environmental factors involved. This condition and its comorbidities have reached epidemic proportions, both in developed and developing countries [1]. Obesity is associated with the development of insulin resistance syndrome (IRS), commonly referred to as metabolic syndrome. IRS is defined by the presence of impaired glucose metabolism, hypertriglyceridemia, low concentrations of serum high-density lipoprotein (HDL) and other alterations associated with an increased risk of cardiovascular disease (CVD). IRS is a key factor in the development of type 2 diabetes mellitus (DM2), CVD and non-alcoholic fatty liver disease (NAFLD) [2]. Clinical management of IRS remains a public health challenge. Indeed, current treatment is based on changes in lifestyle through hygienic-dietary measures (diet and exercise) combined with medical therapy, if necessary, to treat the components of IRS separately and not as a whole entity.

In recent years, it has become clear that the alteration of the intestinal microbiota composition can contribute to the development of insulin resistance associated with obesity [3–5]. In this sense, a decreased *Bacteroidetes*/*Firmicutes* ratio has been described in obese compared to lean individuals [6]. Furthermore, an aberrant intestinal microbiota is able to promote a state of low-grade systemic inflammation, insulin resistance and increased CVD risk through mechanisms that include exposure to bacterial products. In particular, lipopolysaccharide (LPS) produces metabolic endotoxaemia capable of modulating pro-inflammatory cytokines and altering glucose and lipid metabolism in the liver and adipose tissue [7, 8]. In addition, it has been reported that high concentrations of serum LPS-binding protein (LBP) are associated with obesity, IRS and DM2 [9].

Probiotics are live microorganisms that confer a health benefit on the host when administered in adequate amounts. They can modulate the gut microbiota and the immune system. The strains most frequently used as probiotics belong to the *Bifidobacterium* and *Lactobacillus* genera [10]. In this regard, our group has reported that the administration of 9 × 10^9^ cfu (colony-forming units)/day of three probiotics strains (*Lactobacillus paracasei* CNCM I-4034, *Lactobacillus rhamnosus* CNCM I-4036 and *Bifidobacterium* breve CNCM I-4035) to healthy subjects during 30 days induced significant colon microbiota modifications [11].

This fact opens the possibility of a new approach in the treatment of IRS, not with drugs but with functional foods containing probiotics [12]. Intervention studies to determine the effect of probiotics on the components of IRS are limited and often contradictory, mainly due to differences in the design of the study protocol and in the selection of the probiotic strain because the effects observed are strain dependent.

In this sense, it has been described that *Lactobacillus reuteri (L. reuteri)* V3401 (CECT 8605) successfully reduces the absorption of cholesterol by intestinal epithelial cells in in vitro and in vivo assays. This cholesterol-absorbing capacity may be induced through an increase in lytic activity specific to *L. reuteri* V3401 [13]. In an animal model, mice fed a hypercholesterolemic diet supplemented with 2 × 10^9^ cfu of *L. reuteri* V3401/day for 57 days exhibited reduced serum cholesterol levels compared to hypercholesterolemic mice that did not receive the probiotic supplement. Moreover, the animals supplemented with the probiotic strain showed glycemic values similar to those of normocholesterolemic mice [13]. These results suggest that this strain might be adequate for the treatment of dyslipidaemia [13]. We therefore hypothesize that *L. reuteri* V3401 may provide a beneficial effect on IRS treatment, and we aim to study *L. reuteri* V3401 effects in a crossover clinical double-blind, randomized, placebo-controlled trial in IRS patients.

## Method/design

### Aim of the study

The main objective is to evaluate the effects of *L. reuteri* V3401 on the composition of intestinal microbiota, anthropometric parameters, and biomarkers of insulin resistance (inflammation, cardiovascular risk, and hepatic steatosis) in IRS patients.

### *Lactobacillus reuteri* V3401 characteristics

Lyophilized *L. reuteri* V3401 capsules will be specially prepared for the trial. The capsules will contain 5 × 10^9^ cfu, in agreement with previous data obtained from studies in animals [13]. The placebo will contain maltodextrin and will also be supplied in capsules. Capsules for the probiotic and placebo will be indistinguishable in shape, colour, and organoleptic conditions, and will be provided by Biosearch Life.

### Study design

**Fig. 1:**
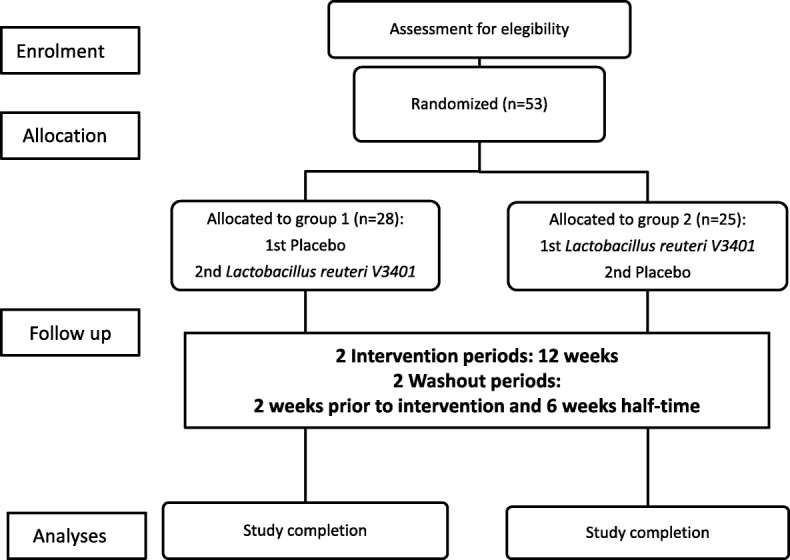
Flow-chart of the study

The study is a randomized, double-blind, crossover, controlled, and single-center trial. The trial will be conducted by members of both the Endocrinology and Nutrition Department and the Gastroenterology Department of Complejo Hospitalario de Jaén (Spain). Participants will be instructed to follow a prebiotic- and probiotic-free diet two weeks before the beginning of the intervention and during the study. The study will be performed as shown in Fig. 1. Blood samples, as well as the food and physical activity questionnaire will be analysed by the members of the University of Granada at the University facilities. Microbial DNA isolated from faecal samples will be sequenced in the facilities of the Hospital Universitario San Cecilio (Granada, Spain).

The protocol (PROSIR version 2) will be approved by the local Ethics Committee of Granada and Jaen (CEI-Granada and CEI Jaén with references CEI- Jaen 25,022,016 and CEI-Granada 28,022,016, respectively), and conducted according to the standards given in the Declaration of Helsinki and the Good Clinical Practice Guidelines [14]. All investigators participating in the trial are appropriately qualified.

The Standard Protocol Items: Recommendations for Interventional Trials (SPIRIT) guidelines for this study protocol has been included as an Additional file 1.

### Participants, interventions, and outcomes

#### Inclusion criteria

Individuals aged 18 to 65 years newly-diagnosed with IRS, according to the criteria of the International Diabetes Federation (IDF), and without any previous treatment for the metabolic syndrome, will participate in the study. The prescription of any treatment not allowed during the course of the study, according to exclusion criteria, will be considered a reason for dropping out. Before starting the trial, written informed consent will be provided by all patients.

#### Exclusion criteria

The use of hypoglycaemic treatment, lipid-lowering medications and treatment with drugs that increase liver enzymes, treatment with specific anti-hypertensives (beta-blockers, angiotensin 2 receptor antagonists, angiotensin-converting enzyme inhibitors) will be considered as an exclusion criteria. Moreover, the exclusion criteria include the presence of kidney disease; diabetes; acute liver injury or severe cirrhosis; immune deficiency conditions; elevated values of C-reactive protein; pregnancy or breastfeeding; history of drug or alcohol abuse; or participation in a study of an investigational medication within the past 30 days.

### Recruitment

A total of 60 subjects are expected to be recruited from the Endocrine and Nutrition Clinic at Complejo Hospitalario de Jaén (Spain) by the physician responsible, through the local newspaper and using advertisement posts at different locations at the hospital facilities.

### Randomization, allocation, and blinding

Patients will be randomly allocated in a 1:1 ratio to receive a capsule containing either lyophilized *L. reuteri* V3401 or placebo once daily with food for 12 weeks. A stratified block-randomization method of a software program for sequence generation will be used. After a 6-week washout, participants will crossover to receive the alternative intervention for another 12 weeks. Subjects and investigators will be blinded to the treatment allocation during the course of the intervention. The blind will be broken following consultation with the sponsor.

The capsules will be prepared in special containers with the random number label. Capsules will be delivered at the beginning of each intervention and six weeks later to ensure probiotic viability.

### Intervention

We will conduct a standard semi-structured clinical interview with clinical assessment. Subsequently, we will audit the clinical reports to extract relevant information (Additional file 2). In addition, a physical examination, and a complete blood test will be also conducted prior to inclusion in the study. Participants meeting the inclusion criteria will be randomly assigned to the probiotic or placebo group. During the intervention, participants will be asked to ingest a capsule per day with food. Capsules will be administered over two periods of 12 weeks. During the washout period, participants will not receive dietary recommendations. Clinical follow-up visits will be scheduled at the beginning, midpoint, and end of each interventional period. At each visit, anthropometric parameters will be registered, and possible adverse effects will be recorded (Table 1).

**Table 1:** Detailed study procedures

Visits (V) Week (W)			V1 (W-2)	V2 (W0)	V3 (W6)	V4 (W12)	Washout	V5 (W18)	V6 (W24)	V7 (W30)
Screening analysis			X							
Demographics/ general information			X							
Informed content form			X							
Randomization and allocation			X							
Treatment distribution			X							
Blood test				X		X		X		X
Stools collection				X	X	X		X	X	X
Hepatic ultrasound				X		X				X
Assessments	General outcomes									
		Clinical interview		X	X	X		X	X	X
		Blood pressure and anthropometry		X	X	X		X	X	X
		3-day dietary record		X		X		X		X
		Food Frequency Questionnaire (FFQ)		X		X		X		X
		Physical activity questionnaire (IPAQ)		X		X		X		X
	Primary outcomes									
		Plasma LPS		X		X		X		X
	Secondary outcomes									
		Blood Pressure		X	X	X		X	X	X
		HOMA		X		X		X		X
		Lipid profile		X		X		X		X
		Inflammatory markers		X		X		X		X
		Hepatic steatosis markers		X		X		X		X

The data and samples will be collected in the Complejo Hospitalario de Jaén by qualified personal, and will be codified according to the biobank of the Public Health System of Andalusia (BSSPA) guidelines. All samples will be managed and processed in accordance with BSSPA protocols. All samples will be centralized at the BSSPA facilities.

### Dietary and physical activity control

Participants will be provided with a list of local foods to avoid due to their potential probiotic or prebiotic content. Subjects will be asked to complete a three-day dietary record at the beginning, midpoint, and end of each intervention period. Energy and dietary intakes will be assessed from the 3-day records using the EvalFINUT software (http://www.finut.org/evalfinut/). Additionally, subjects will complete the Food Frequency Questionnaire (FFQ) at the beginning of each intervention period. Participants will receive nutritional counselling on a healthy Mediterranean diet providing approximately 25 kcal/kg/ day to achieve and maintain 7% loss of initial body weight and increase moderate-intensity physical activity (such as brisk walking) for at least 150 min/week following the guidelines of the American Diabetes Association for the prevention or delay of DM2 [15]. Likewise, physical activity will be monitored through the International Physical Activity Questionnaire (IPAQ) at the beginning, midpoint, and end of each intervention period. In the following visits, an intensive lifestyle intervention programme will be carried out to reinforce the behaviour.

### Outcome measures

Clinical and anthropometric parameters (weight, height, waist circumference and blood pressure) will be recorded. A hepatic ultrasound (US) test will also be performed at the beginning of the two intervention periods, as well as at the end of the intervention periods. US diagnostic criteria for diagnosis and grading of hepatic steatosis will be used [16]. Smoking habits and anxiolytic and antihistaminic drug intake will also be recorded.

Blood samples will be collected after 12 h of fasting at the beginning and at the end of each intervention period (t0, t12, t18 and t30). Stool samples will also be taken at baseline and at 6, 12, 18, 24, and 30 weeks (t0, t6, t12, t18, t24, and t30, respectively). Serum will be collected by centrifugation of blood samples for the determination of biochemical and inflammation biomarkers. Faecal samples will be collected and kept at − 80 °C until analysis. All samples will remain frozen at − 80 °C in the BSSPA Platform.

#### Primary outcome

The primary outcome will be the change in plasma lipopolysaccharide (LPS) concentration. The determination of LPS concentration and LPS-binding lipoprotein (LBP) will be performed by a simple ELISA (Cloud-Clone Corp., Houston, USA).

#### Secondary outcomes

Body Mass Index (BMI) and waist circumference will be registered at the beginning, midpoint, and end of each intervention period by qualified staff. Systolic and diastolic blood pressures will be determined three times at 5-min intervals while volunteers are seated using a standardized mercury sphygmomanometer; the final two measurements will be averaged for the analysis.

Serum glucose, insulin, Homeostasis Model Assessment (HOMA) index, cholesterol, LDL, HDL, triacylglycerols, glutamate-pyruvate transaminase (GPT), glutamate-oxaloacetate transaminase (GOT), gamma-glutamyltransferase (γ-GT), glycated haemoglobin, and C-reactive protein will be determined before and at the end of each intervention period by standard methods.

Inflammatory and CDV biomarkers, including adiponectin, resistin, myeloperoxidase (MPO), plasminogen activator inhibitor-1 (PAI-1), tumour necrosis factor-alpha (TNF-α), interleukin-6 (IL-6), interleukin-8 (IL-8), soluble intercellular adhesion molecule-1 (sICAM-1) and soluble vascular adhesion molecule-1 (sVCAM-1), will be measured using MILLIPLEXMAP test kits (Milliplex Map Kit, Millipore Corp, Billerica, MA). Arginase, prolidase and retinol binding protein-4 will be measured as hepatic steatosis markers. The arginase and prolidase activity will be measured according to Palomero-Rodriguez et al. [17] and Kayadibi et al. [18], respectively, whereas RBP-4 will be determined by ELISA technology (AdipoGen Inc).

For the study of intestinal microbiota composition, a 16S metagenomics sequencing will be performed. In brief, faecal DNA will be extracted using a commercial isolation kit (QIAamp DNA Stool Mimi Kit, Qiagen). The variable V3 and V4 regions of the 16S rRNA gene will be PCR-amplified and sequenced on an Illumina MiSeq platform at Hospital Universitario San Cecilio, Spain, following the Illumina recommendations.

### Withdrawal, dropout, discontinuation, and compliance

Withdrawal will be allowed at any time during the trial. Participants may be advised to discontinue the trial if there is no compliance or if a severe adverse event occurs. Women will be advised not to become pregnant during the study. Participants will be instructed to return the unconsumed capsules when collecting the follow-up doses. Participants whose compliance with treatments or placebo is ≤80% of the total, those who become pregnant and those who develop diabetes will be considered dropouts.

### Adverse events and safety monitoring

All *Lactobacillus* strains are considered safe by the EFSA (European Food Safety Authority). However, each participant will be monitored for abdominal discomfort, diarrhoea, bloating, constipation and nausea/vomiting, among other possible effects, during each visit, and all unexpected adverse events will be reported by participants and written on the individual case report form by the investigator.

### Sample size and power analysis

Based on the range and median value of LPS [8] and assuming a power of 80% and a significance level of 5%, the minimum number of subjects will be 21 per arm. To avoid possible bias caused by gender and taking into account withdrawal, we will be recruiting a total of 60 subjects. Missing data will be considered as unavailable data.

### Statistical analysis

Data analyses will be performed at the end of the intervention, therefore, a replacement or substitution of a withdrawal subject is not planned; therefore, the missing data will be considered unavailable data.

The normality of variables will be assessed. Variables not following a normal distribution will be transformed. Normal variables will be expressed as the mean and standard error of the mean. An intention-to-treat analysis will be performed for the analysis of efficacy. In order to determine differences in basal characteristics, a paired *t-test* will be used. Carryover effects will be assessed by three-way ANOVA. A general lineal model for repeated measurements will be performed to evaluate differences between variables at the beginning, midpoint, and end of each intervention period. Two-way ANOVA will be used to determine the influence of treatment (probiotic or placebo) and time on the continuous dependent variables. Statistical analyses will be performed using IBM SPSS statistics v22.

## Discussion

The present study has been designed to demonstrate whether a probiotic strain, *L. reuteri* V3401, is capable of reducing the IRS components together with healthy lifestyle recommendations (hypocaloric diet and physical activity).

Currently, altered intestinal microbiota is considered a new player in IRS based on its capacity to promote a state of low-grade systemic inflammation, insulin resistance and increased cardiovascular risk through mechanisms that include exposure to bacterial products such as LPS, a component of the outer membrane of gram-negative bacteria. It is currently accepted that the increase in serum levels of LPS produces a metabolic endotoxaemia capable of modulating pro-inflammatory cytokines, as well as glucose and lipid metabolism in the liver and adipose tissue. There is scientific evidence to support the contribution of LPS in DM2 and obesity [7]. Additionally, on the basis of studies in both animals and humans, dietary intake appears to be a major regulator of the structure and function of the gut microbiota [19]. Therefore, promoting changes in the gut microbiota combined with an adequate Mediterranean diet might play an important role in ensuring the efficacy and success of reducing IRS components and avoiding DM2 development.

However, a relatively small number of randomized, clinically controlled studies assessing the beneficial effects of probiotics on metabolic syndrome parameters have been reported. When looking for a specific clinical response, special care must be taken when selecting the strain because probiotic effects are strain specific [12]. The effects of probiotics are explained by their contribution to the intestinal microbiota composition, maintenance of gut barrier function and their immunomodulation capacity [10, 12].

As mentioned above, the most commonly used strains of probiotics are *Bifidobacterium* and *Lactobacillus* spp*.* The consumption of *L. acidophilus* NCFM has been described to preserve insulin sensitivity without affecting systemic inflammation [20]. Furthermore, *L. reuteri* LR6 has been shown to decrease total cholesterol values and increase HDL-cholesterol levels in the plasma of rats fed a hypercholesterolemic diet [21]. In humans, enrichment of gut microbiota with *L. reuteri* SD5865 has been reported to increase insulin secretion, possibly due to augmented incretin release, although this strain does not seem to affect insulin sensitivity or body fat distribution [22]. However, in a correlation study, Million et al. detected *L. reuteri* in 20% of their study population, with occurrence increasing along with BMI values (7, 8, 34 and 22% for anorexic, lean, overweight and obese individuals, respectively) [23].

To the best of our knowledge, no studies have tested the possible beneficial effect of *L. reuteri* V3401 in patients with IRS. Based on the background provided above, we propose to study the *L. reuteri* V3401 strain by means of an intervention study in subjects with IRS who will follow a healthy lifestyle (hypocaloric diet and physical activity).

## Additional files

Additional file 1: SPIRIT 2013 Checklist: Recommended items to address in a clinical trial protocol and related documents. (DOC 120 kb)

Additional file 2: Clinical interview guide. (DOC 27 kb)

## Abbreviations

BMI: Body Mass Index
Cfu: Colony forming units
CVD: Cardiovascular disease
DM2: Diabetes mellitus 2
EFSA: European Food Safety Authority
FFQ: Food Frequency Questionnaire
GOT: Glutamate-oxalacetate transaminase
GPT: Glutamate-pyruvate transaminase
HDL: Serum high-density lipoprotein
HOMA: Homeostasis Model Assessment
IDF: International Diabetes Federation
IL-6: Interleukin-6
IL-8: Interleukin-8
IPAQ: International Physical Activity Questionnaire
IRS: Insulin resistance syndrome
LBP: Serum LPS binding protein
LPS: Lipopolysaccharide
MPO: Myeloperoxidase
NAFLD: Non-alcoholic fatty liver disease
PAI-1: Plasminogen activator inhibitor-1
sICAM-1: Soluble intracellular adhesion molecule-1
sVCAM-1: Vascular adhesion molecule-1
TNF-α: Tumor necrosis factor-alpha
US: Ultrasound
γ-GT: Gamma-glutamyltransferase

## References

[1] Kushner RF, Kahan S (2018). Introduction: the state of obesity in 2017. Med Clin North Am.

[2] Alberti KGMM, Eckel RH, Grundy SM, Zimmet PZ, Cleeman JI, Donato KA, Fruchart JC, James WP, Loria CM, Smith SC (2009). Harmonizing the metabolic syndrome: a joint interim statement of the international diabetes federation task force on epidemiology and prevention; National Heart, Lung, and Blood Institute; American Heart Association; world heart federation; international atherosclerosis society; and International Association for the Study of obesity. Circulation.

[3] Bäckhed F, Ding H, Wang T, Hooper LV, Koh GY, Nagy A (2004). The gut microbiota as an environmental factor that regulates fat storage. Proc Natl Acad Sci U S A.

[4] Bäckhed F, Manchester JK, Semenkovich CF, Gordon JI (2007). Mechanisms underlying the resistance to diet-induced obesity in germ-free mice. Proc Natl Acad Sci.

[5] Turnbaugh PJ, Ley RE, Mahowald MA, Magrini V, Mardis ER, Gordon JI (2006). An obesity-associated gut microbiome with increased capacity for energy harvest. Nature.

[6] Turnbaugh PJ, Hamady M, Yatsunenko T, Cantarel BL, Duncan A, Ley RE, Sogin ML, Jones WJ, Roe BA, Affourtit JP, Egholm M, Henrissat B, Heath AC, Knight R, Gordon JI (2009). A core gut microbiome in obese and lean twins. Nature.

[7] Cani PD, Amar J, Iglesias MA, Poggi M, Knauf C, Bastelica D, Neyrinck AM, Fava F, Tuohy KM, Chabo C, Waget A, Delmée E, Cousin B, Sulpice T, Chamontin B, Ferrières J, Tanti JF, Gibson GR, Casteilla L, Delzenne NM, Alessi MC, Burcelin R (2007). Metabolic Endotoxemia initiates obesity and insulin resistance. Diabetes.

[8] Lassenius MI, Pietiläinen KH, Pussinen PJ, Syrjänen J, Forsblom C, Pörsti I, Rissanen A, Kaprio J, Mustonen J, Groop PH, Lehto M (2011). Bacterial endotoxin activity in human serum is associated with dyslipidemia insulin resistance, obesity, and chronic inflammation. Diabetes Care.

[9] Sun L, Yu Z, Ye X, Zou S, Li H, Yu D, Wu H, Chen Y, Dore J, Clément K, Hu FB, Lin X (2010). A marker of endotoxemia is associated with obesity and related metabolic disorders in apparently healthy Chinese. Diabetes Care.

[10] Bermudez-Brito M, Plaza-Díaz J, Muñoz-Quezada S, Gómez-Llorente C, Gil A (2012). Probiotic mechanisms of action. Ann Nutr Metab.

[11] Plaza-Díaz J, Fernández-Caballero JÁ, Chueca N, García F, Gómez-Llorente C, Sáez-Lara MJ, Fontana L, Gil Á (2015). Pyrosequencing analysis reveals changes in intestinal microbiota of healthy adults who received a daily dose of immunomodulatory probiotic strains. Nutrients.

[12] Miglioranza Scavuzzi B, Miglioranza LH d S, Henrique FC, Pitelli Paroschi T, Lozovoy MAB, Simão ANC, Dichi I (2015). The role of probiotics on each component of the metabolic syndrome and other cardiovascular risks. Expert Opin Ther Targets.

[13] Sañudo Otero AI, Criado García R, Rodríguez Nogales A, Garach Domech A, Olivares Martín M, Gálvez Peralta JJ, De La Escalera Hueso S, Duarte Pérez JM, Zarzuelo Zurita A, Bañuelos Hortigüela O. Probiotic strains having cholesterol absorbing capacity, methods and uses thereof. Google Patents; 2016. https://encrypted.google.com/patents/EP3031930A1?cl=en&hl=es Accessed 14 Jan 2018.

[14] Vijayananthan A, Nawawi O (2008). The importance of good clinical practice guidelines and its role in clinical trials. Biomed Imaging Interv J.

[15] American Diabetes Association (2018). 5. Prevention or Delay of Type 2 Diabetes: Standards of Medical Care in Diabetes-2018. Diabetes Care.

[16] Csendes P, Paolinelli P, Busel D, Venturelli V, Rodríguez J (2004). Higado graso: Ultrasonido y correlación anatomopatológica. Rev Chil Radiol.

[17] Palomero Rodríguez MA, García Navas R, Laporta Báez Y, Al Kassam Martínez D, de Vicente Sánchez J, Cacharro Moras LM, Sánchez Conde P, Mollinedo F, Muriel Villoria C (2012). Relationship between arginase activity and the storage time of packed red blood cells. Rev Esp Anestesiol Reanim.

[18] Kayadibi H, Gültepe M, Yasar B, Ince AT, Ozcan O, Ipcioglu OM, Kurdas OO, Bolat B, Benek YZ, Guveli H, Atalay S, Ozkara S, Keskin O (2009). Diagnostic value of serum prolidase enzyme activity to predict the liver histological lesions in non-alcoholic fatty liver disease: a surrogate marker to distinguish steatohepatitis from simple steatosis. Dig Dis Sci.

[19] Lynch SV, Pedersen O (2016). The human intestinal microbiome in health and disease. N Engl J Med.

[20] Andreasen AS, Larsen N, Pedersen-Skovsgaard T, Berg RM, Møller K, Svendsen KD, Jakobsen M, Pedersen BK (2010). Effects of lactobacillus acidophilus NCFM on insulin sensitivity and the systemic inflammatory response in human subjects. Br J Nutr.

[21] Singh TP, Malik RK, Katkamwar SG, Kaur G (2015). Hypocholesterolemic effects of *Lactobacillus reuteri* LR6 in rats fed on high-cholesterol diet. Int J Food Sci Nutr.

[22] Simon MC, Strassburger K, Nowotny B, Kolb H, Nowotny P, Burkart V, Zivehe F, Hwang JH, Stehle P, Pacini G, Hartmann B, Holst JJ, MacKenzie C, Bindels LB, Martinez I, Walter J, Henrich B, Schloot NC, Roden M (2015). Intake of lactobacillus reuteri improves incretin and insulin secretion in glucose-tolerant humans: a proof of concept. Diabetes Care.

[23] Million M, Angelakis E, Maraninchi M, Henry M, Giorgi R, Valero R, Vialettes B, Raoult D (2013). Correlation between body mass index and gut concentrations of lactobacillus reuteri, Bifidobacterium animalis, Methanobrevibacter smithii and Escherichia coli. Int J Obes Nat.

