# Supplementary material for: Evaluation of the effect of Lactobacillus reuteri V3401 on biomarkers of inflammation, cardiovascular risk and liver steatosis in obese adults with metabolic syndrome: a randomized clinical trial (PROSIR)
Source: BMC Complement Altern Med. 2018 Nov 20;18:306. doi: 10.1186/s12906-018-2371-x (PMC6245703; doi:10.1186/s12906-018-2371-x)
Supplement: Supplementary file 2 — Clinical interview guide. (DOC 27 kb) [file 12906_2018_2371_MOESM2_ESM.doc]

**Additional file 2. Clinical Interview guide**

1. Family medical history.

2. Past medical and surgical history.

3. Social history.

4. Medication and allergies.

5. Alcohol consumption and tobacco use.

6. Eating habits, in particular:

-snacking highly processed and calorie-rich foods.

-large food portions.

-skipping breakfasts.

-eating out.

-”emotional eating”

7. Physical activity.
